# Health promotion for mild frailty based on behaviour change: Perceptions of older people and service providers

**DOI:** 10.1111/hsc.12781

**Published:** 2019-05-31

**Authors:** Christina Avgerinou, Benjamin Gardner, Kalpa Kharicha, Rachael Frost, Ann Liljas, Rekha Elaswarapu, Jill Manthorpe, Vari M. Drennan, Claire Goodman, Steve Iliffe, Kate Walters

**Affiliations:** ^1^ Department of Primary Care and Population Health University College London London UK; ^2^ Department of Psychology King’s College London London UK; ^3^ Age UK Ealing (Patient and Public Involvement and Engagement representative) London UK; ^4^ Social Care Workforce Research Unit King’s College London London UK; ^5^ Centre for Health and Social Care Research Kingston University and St George’s, University of London London UK; ^6^ Centre for Research in Primary and Community Care University of Hertfordshire Hatfield UK

**Keywords:** behaviour change techniques, frailty, goal setting, health promotion, qualitative study

## Abstract

Mild frailty is common among older people, but it is potentially reversible with health promotion interventions. Behaviour change may be a key to preventing progression of frailty; however, we know little about what interventions work best and how a behaviour change approach would be perceived by this group. The aim of this study was to explore how mildly frail older people perceive health promotion based on behaviour change and what factors affect engagement with this approach. We conducted semi‐structured interviews with 16 older people with mild frailty who received a pilot home‐based behaviour change health promotion service, including a dyad of older person/family carer, and two service providers delivering the service in two diverse areas of South England. Interviews were audio‐recorded, transcribed and thematically analysed. The concept of goal setting was acceptable to most participants, though the process of goal setting needed time and consideration. Goals on maintaining independence, monitoring of progress and receiving feedback were reported to increase motivation. Physical/mental capability and knowledge/perception of own needs were main determinants of the type of goals chosen by participants as well as the approach used by the project workers. Older people with complex needs benefited from care coordination, with a combination of goal setting and elements of social, practical and emotional support in varying proportions. Mildly frail older people responded well to a behaviour change approach to promote health and well‐being. Further consideration is needed of the most effective strategies based on complexity of needs, and how to overcome barriers among people with cognitive impairment.

**What is known about the topic**

- Older people with mild frailty engage in activities to promote health and well‐being, although ill health, social and environmental factors are potential barriers to maintaining independence.
- Behaviour change techniques are key components of health promotion interventions and may be helpful in preventing progression of frailty.

**What this paper adds**

- Findings from this qualitative study show that goal setting was perceived as a positive experience by the majority of mildly frail older people receiving a health promotion service based on behaviour change.
- Maintaining independence was a priority.
- Monitoring of progress and receiving feedback increased motivation.

## 1 INTRODUCTION

Frailty is an age‐related condition characterised by loss of biological reserves across multiple organ systems, and vulnerability to physiological decompensation after a stressor event (Clegg, Young, Iliffe, Rikkert, & Rockwood, 2013). It is associated with an increased risk of hospitalisation, falls, moves to care homes and death (Ensrud et al., 2008; Rockwood et al., 2004). Frailty affects approximately 11% of people aged above 65 in developed countries, with approximately 41% considered pre‐frail based on a systematic review of 15 studies from European countries, United States, Canada, Australia and Taiwan (Collard, Boter, Schoevers, & Oude Voshaar, 2012). Pre‐frailty (mild frailty) is an intermediate state between being frail and robust (Fried et al., 2001). Older people with mild frailty can recover after a stressor event (e.g. minor injury, infection, new medication), but may increasingly rely on others for help with instrumental activities of daily living (IADLs) (Rockwood et al., 2005). Frailty is potentially reversible (Ng et al., 2015); complex interventions can reduce the risk of moving to care homes, hospital admission and falls in frail older people (Beswick et al., 2008). Although interventions targeted at mild frailty can potentially prevent frailty progression, there is insufficient evidence to recommend specific interventions (Frost et al., 2017).

A deficit model has traditionally been the main focus of available evidence informing health policies, the main disadvantage of which is it may be potentially disempowering for older people. As opposed to the deficit model, the asset model draws on the theory of salutogenesis (Antonovsky, 1996) to investigate key ‘health assets’ that support the creation of health rather than the prevention of disease (Morgan & Ziglio, 2007). According to Baltes' theory, successful ageing is the result of maintaining activities prioritised by the older person and their environment (selection), optimising the performance of these (optimisation) and compensating for limitations (compensation) (Baltes & Baltes, 1990).

Behaviour change is a key to health promotion. Modifiable behaviours such as exercise (Clegg, Barber, Young, Forster, & Iliffe, 2012; Liu & Latham, 2009) and diet (Kojima, Avgerinou, Iliffe, & Walters, 2018) have potential to alter the progression of frailty. Promoting health behaviour change depends on identifying strategies most likely to have positive impacts. According to the COM‐B framework, all behaviour depends on three essential components: Capability, Opportunity and Motivation (Michie, Stralen, & West, 2011). Capability to perform the behaviour can be either ‘physical’ (having the physical skills, strength or stamina) or ‘psychological’ (having the knowledge, psychological skills, strength or stamina). Opportunity is defined as “all the factors that lie outside the individual and make the behaviour possible or prompt it” (Michie et al., 2011, p. 4). Opportunity can be either ‘physical’ (what the environment allows or facilitates in terms of time, triggers, resources, locations, physical barriers, etc.) or ‘social’ (including interpersonal influences, social cues, cultural norms). Motivation is defined as “all those brain processes that energise and direct behaviour, not just goals and conscious decision‐making” (Michie et al., 2011, p. 4). Motivation can be ‘reflective’ (involving self‐conscious planning and evaluations) or ‘automatic’ (processing needs and desires, impulses and reflex responses) (Michie, Atkins, & West, 2014, pp. 59–60). Around this hub of capability, opportunity and motivation are situated nine intervention functions (broad category of means by which interventions may change behaviour): education, persuasion, incentivisation, coercion, training, enablement, modelling, environmental restructuring and restrictions (Michie et al., 2011).

Our systematic review described existing home‐based health behaviour change interventions for older people with frailty or at risk of frailty, and examined links between their content and their potential to initiate behaviour change and improve outcomes (Gardner et al., 2017). Drawing on recent developments in behavioural science, we classified intervention content according to the function(s) performed by the intervention and component behaviour change techniques (BCTs). BCTs are the irreducible intervention components that serve to perform one or more functions, such as setting goals or monitoring behaviour (Michie et al., 2013). The BCTs ‘provide instruction on how to perform a behaviour’, ‘add objects to the environment’ and ‘restructure the physical environment’, and the intervention functions education and enablement showed most potential for improving physical function (Gardner et al., 2017). This review revealed the lack of attention to behaviour change within intervention design, and to outcomes and mechanisms by which these complex interventions might work.

Despite several studies reporting older people's perceptions of health promotion interventions targeting specific problems, such as falls (Bunn, Dickinson, Barnett‐Page, McInnes, & Horton, 2008; McMahon, Talley, & Wyman, 2011), smoking (Kerr, Watson, Tolson, Lough, & Brown, 2006), or reducing sedentary behaviour (Heseltine et al., 2015), very few studies explore how older people experience the delivery of BCTs in particular, within generic health promotion. A better understanding of older people's attitudes to and experiences of behaviour change would shed light on which strategies are considered most effective, and which techniques older people best engage with. A critical exploration of reasons for non‐engagement can also contribute to improving the delivery of BCTs and hence maximise older people's benefits from health promotion interventions. This paper reports on a study that explored how health promotion based on behaviour change is perceived by both recipients and providers of a service, to understand how BCTs are received, which factors affect engagement with behaviour change strategies, and whether adjustments are needed for mildly frail older people.

## 2 DESIGN AND METHODS

### 2.1 Study design and setting

This qualitative study was nested within a feasibility randomised controlled trial of a theory‐ and evidence‐based home‐based health promotion service for older people with mild frailty (HomeHealth) funded by Health Technology Assessment, National Institute for Health Research (Walters et al., 2017). As part of the mixed‐methods evaluation of the service, we conducted qualitative interviews with intervention participants and service providers following the completion of the feasibility trial.

### 2.2 The HomeHealth service

HomeHealth was a prototype of a new home‐based health promotion service for older people with mild frailty, developed using a co‐design approach and tailored to the individual's needs (Walters et al., 2017). The service was designed using the COM‐B behaviour change theory (Michie et al., 2011) and was based on the principle of maintaining assets (Morgan & Ziglio, 2007). This was refined through one‐to‐one meetings with commissioners, managers and practitioners in urban and semi‐rural areas and service development panels with frailer older people, health/socialcare and voluntary sector professionals, commissioners (funders), policy makers, academic experts and public representatives. The service was delivered in five to six appointments over a period of 6 months. The role of HomeHealth Project Worker was created for the purposes of the study. Criteria for the recruitment of project workers were excellent communication skills and previous experience of working with older people in community settings. The two project workers received training in communication skills, physical activity and exercise, nutrition and behaviour change, with weekly supervision by an expert in older people and communication skills. The service was targeted at addressing four key domains: mobility, nutrition, socialising and psychological well‐being, as well as other topics raised by participants. The project workers delivered tailored education, enablement, training and environmental restructuring as the main intervention functions. The core set of BCTs used included goal setting, action planning and monitoring progress, maintenance of behaviours and developing habits. Goals were divided into: (a) an outcome goal (i.e. the overarching goal that the older person would like to achieve); (b) behavioural goal(s) (i.e. the specific action(s) agreed with the older person to achieve their outcome goal); and (c) SMART goals (detailed action plans that specify when, where, what and with whom the behavioural goal will be achieved). SMART goals are specific, measurable (recordable whether the goal was met or not), achievable (to build motivation), relevant (important to the person) and timely (achievable within a target time). The logic model of the HomeHealth intervention is published elsewhere, as have data relating to the practical and organisational aspects of delivering the HomeHealth service, including fidelity of delivery (Walters et al., 2017).

### 2.3 Participant sampling, recruitment and procedure

We undertook face‐to‐face semi‐structured qualitative interviews with older people who had received the HomeHealth service, and the two project workers who delivered the service. Participants were recruited from four different NHS general practices (two in urban and two in semi‐rural areas) in London and Hertfordshire, UK. Eligibility criteria for entry into the HomeHealth trial were as follows: aged ≥65 registered with a participating general practice, scoring as mildly frail on the Clinical Frailty Scale (Rockwood et al., 2005) (i.e. evident slowing, needing help in high order IADLs (finances, transportation, heavy housework, medications), progressively impaired ability to shop, walking outside alone, meal preparation and housework), community‐dwelling (including extra care housing), a life expectancy of >6 months, and having capacity to consent. A list generated through electronic primary care searches based on the above criteria was further screened by the GP for exclusions, and a random sample of participants was invited by post to take part in the study. At a second stage, interested participants were screened for eligibility over the phone by a researcher using a set of questions based on the Fried frailty criteria (Fried et al., 2001), asking them about slowness, fatigue and if they needed assistance with ADLs. Baseline assessment was carried out at home, informed consent was sought and eligible participants entered the trial (Walters et al., 2017).

The mean age of participants recruited to the trial was 80 years at baseline. Baseline measurements showed that the sample was independent in ADLs based on Modified Barthel Index, but were demonstrating some signs of frailty with a lower grip strength and slower gait speed than the population average, and having an average of three to four long‐term health conditions (Walters et al., 2017).

The additional criterion for entry into the present interview study was that participants must have received the HomeHealth service as part of the HomeHealth intervention trial. All 26 people who had received the service were invited to take part, and 16 consented to interview. Participants were interviewed individually, except one older person with dementia who was interviewed with their family carer. Eighteen interviews were conducted (16 with older people, including a dyad of an older person and their carer and two project workers). Participant characteristics are presented in Table 1.

**Table 1:** Demographics of older people interviewed (*n* = 16)

Characteristic	Category	*N*
Area ‐ type	Urban	11
	Semi‐rural	5
Gender	Male	3
	Female	13
Age group (years)	70–74	2
	75–79	4
	80–84	4
	85–89	3
	90+	3
Ethnicity	White British	12
	Any other White background	3
	Black African	1
Age at end of formal education	Before 15 years	2
	Between the age of 15 and 16 years	4
	Between 17 and 20 years	2
	21 years and over	8

All interviews followed the end of service delivery (August–December 2016). Topic guides were developed with input from the research team and refined iteratively as the study progressed, exploring participants' experiences of the service, motivation, identification of goals, how they found use of BCTs, and perceived impact of the service. Semi‐structured interviews were broader in scope, but we have only extracted data relevant to our present research questions in this paper, as other data regarding fidelity have been reported elsewhere. Interviews with older people (conducted by CA or KK) took place in participants' homes with written informed consent. Participants were given a £20/$28 high street shopping voucher as thanks. Service provider interviews were conducted by JM in a private university office. Interviews were audio‐recorded, transcribed verbatim and data were anonymised. The mean length of the interview was 49 min (range 23–87 min).

### 2.4 Data analysis

We undertook thematic analysis of data using a six‐stage process, including familiarisation with data, data coding, searching for themes, reviewing themes, defining and naming themes, and producing the report (Braun & Clarke, 2006). The multi‐disciplinary analysis team (CA, KW, KK, RF, AL, RE) independently read transcripts and inductively identified a preliminary thematic framework to guide further coding. This thematic framework was applied to a selection of transcripts by CA, further refined by the team and then applied by CA to all transcripts. COM‐B and behaviour change theory were used to inform the analysis within a primarily inductive approach. The themes generated were then considered and interpreted by the team. NVivo software was used to manage the data (NVivo 11 Pro for Windows, QSR International). In this paper, we report on themes focusing on how older people with mild frailty experienced the behaviour change approach.

### 2.5 Ethical approval

The study was approved by the NHS Camden and King's Cross Research Ethics Committee (ref. 14/LO/1698). All participants provided informed consent to participate in the study and anonymity was assured. Confidentiality was also assured subject to agreement that potential concerns about safeguarding/ill treatment of a vulnerable adult would be raised to appropriate authorities.

## 3 FINDINGS

We identified three main themes: (a) factors affecting choice of goals and type of approach used; (b) factors affecting participants' engagement with behaviour change; (c) overall perceptions of goal setting and other BCTs.

### 3.1 Factors affecting choice of goals and type of approach used

#### 3.1.1 Physical and mental health status

The degree of physical and mental capability was the main determinant of the type of goals chosen by participants as well as the approach used by the project workers. More specifically, people who experienced fatigue/low energy, poor balance or restricted mobility chose behavioural goals of undertaking home exercise, starting to walk or increasing their walking and joining a class. Provision of exercise aids and demonstration leaflets was well‐received, and monitoring of performance including self‐monitoring helped participants to sustain physical activity:

My balance is much improved, because she gave me or suggested exercises I could do. She gave me some literature. She covered most exercises I should do and I did. And I felt much more positive, yes. Those were a tremendous, tremendous help. (OP16, Male, 87 years)

Those who were frailer and found using ordinary public or private transport difficult considered the information provided by the project workers about options such as taxi cards (publicly funded travel subsidy for disabled people) and ‘blue badges’ (scheme allowing car parking allocations for disabled people) to be useful:

I got a lot of help one way or another, often from people that (project worker) put me in touch with, you know, the right experts. And that was really helpful. I mean I didn't know about the taxi card, for example, which, if you don't mind it being a bit late, is brilliant. (OP4, Male, 78 years)

Some participants reported restructuring the home environment either by themselves (e.g. de‐cluttering) or with help from occupational therapy services in making adaptations such as bathroom adjustments, fitting of grab rails and taking up of other advice about equipment, all of which were well‐received.

Psychological capability was often affected by loneliness and life events such as widowhood and bereavement, leading to increased wish for support:

Because I was at a very low point and I was crying a lot. I felt really down and I was, I don’t know how, but somebody said that they help you inasmuch as they can, … suggest places to go and things to do, so you’re not so isolated, because you tend to isolate yourself. (OP12, Female, 73 years)

Examples of associated behavioural goals included contacting mental health services, engaging with physical activity, taking up leisure activities, phoning a befriending service, creating time for themselves, engaging in voluntary activities in the community or sharing previous experiences as carers/caregivers.

Cognitive functions were an important element of the older person's capability. During the feasibility trial, one participant was diagnosed with mild cognitive impairment and one with dementia, after referral from project workers to the memory clinic. Cognitive decline was a barrier to engagement with goal setting, because of: (a) poor memory, which made the person with dementia unable to remember the appointments (although the person with mild cognitive impairment could keep a diary and recall appointments) and (b) declining executive skills leading to inability to set goals and keep to a plan:

I would have said to her, ‘Do you think you’re going to remember to do the exercises?’ But I think that wouldn’t have worked with this participant. So I gave her the exercise equipment just to do once, maybe once a week. And, when I came back, she said she had forgotten. (Project worker, area A)

In both cases, the project worker provided practical (e.g. support in facilitating environmental changes) and emotional support (e.g. arranging for a family member to accompany the older person to exercise classes) to enable goals to be met. Despite the initial challenges of identifying and responding to impaired cognition, the project worker felt that interaction with this client had been rewarding:

In this case it was luck that her daughter came in just halfway through the second appointment. And we were able to sort of have an agreement that we would all work together, that it was okay to remind this participant of the appointment. And that worked. (Project worker, area A)

#### 3.1.2 Information and perception of own needs

Knowledge about different topics played a role in how older people prioritised interventions. Provision of information by project workers was generally appreciated. It could form an important part of an overall comprehensive approach – for example in supporting an older person with caring responsibilities, education, problem‐solving and help in coping with setbacks prompted her to make practical changes and helped her to cope better:

Chatting with (project worker) it made me think more positive … we changed the car, got an estate (car). He's got a mobility scooter. He's got a wheelchair that I can take him about in. And it made us think, ‘Well, hang on, we can, I can cope with this,’ which I wasn't doing very well at the beginning. (OP6, Female, 72 years)

However, information needed to be perceived as relevant to the older person's needs and tailored to their situation. One of the project workers thought that lack of knowledge and education was the main reason why undernutrition was not perceived as a problem:

It's really, really difficult trying to get somebody to want to gain weight, because it's so unknown. People really don't understand the consequences of malnutrition and often don't think they're malnourished. (Project worker, area A)

When the intervention did not fit with the older person's views of what was healthy, there was evident cognitive dissonance. The example of nutrition highlighted how suggestions to change eating habits were resisted, because (unlike the earlier examples of needing to maintain independence) this was not a recognised problem and so people were not motivated to make changes in this area:

I did a food diary. I was told I was malnourished after that, which I thought was rather funny … I think I eat perfectly well. I don’t know what they were talking about. (OP4, Male, 78 years)

…when I suggested is he eating enough, he found that really difficult to grasp. And I said, ‘And how are your clothes feeling? I don't know what is normal for you.’ And he'd say, ‘They are baggier, but, you know, I'm eating a healthy, balanced diet, so I don't see what the problem is.’ … If somebody says they're fine and they don't want to work on it, there's a barrier there. (Project worker, area A)

### 3.2 Factors affecting participants' engagement with behaviour change

#### 3.2.1 Sources of motivation

Motivation to remain independent appeared to be an important determinant affecting engagement with a behaviour change approach. Independence was represented as the ability to live without the assistance of other people. This was reported as the main desired outcome for many participants. Being able to still drive and get out was evidence of maintaining this ability, countering fears of losing independence and being seen as needing to move to long‐term care:

That’s the most important thing for me. Yes, to be able to go out and also to be able to drive. Yes, because it’s not easy taking her (dog) on the lead, with a crutch. So, if I can drive to such a lot of nice places near, within a mile or two, and let her straight off her lead and we both enjoy it. (OP13, Female, 92 years)

One of the extrinsic motivating factors most commonly reported by older people was the project worker, who was perceived as the main incentive and enabler:

She actually got me walking in the afternoons. It was only my laziness that stopped me normally walking in the afternoons since the tendonitis … I think she did a good job there. (OP1, Male, 82 years)

Adverse personal circumstances around the time of delivery of the service (e.g. worsening disability of a spouse) could improve motivation through greater need for help and support. However, circumstances such as deteriorating physical health (e.g. arthritis, multiple medical appointments following hospital discharge) could affect people's physical capability to reach outcome goals. One participant felt that progress towards psychological well‐being goals was hindered by them being busy selling their property, and another was discouraged from volunteering by limited familiarity with technology.

Intrinsic motivation was rarely discussed by older people, although one thought their lack of confidence was a barrier. Some found it difficult to initiate socialising goals. Deep‐rooted psychological factors prevented some from taking the first step:

That was the difficult thing to do … , to actually meet up with complete strangers. … I'll never forget it. … you say ‘hello’ and… you want to join and fill out a form. And then… somebody else comes along and you just sort of say ‘hello’ … then you sort of start off and then you start talking to everyone. It's really nice … You didn't feel like you were on your own. (OP12, Female, 73 years)

One project worker thought people had to be driven by an internal need to engage with the process and make changes:

Ultimately it's down to them, because, you know, they have to want to do it for themselves at the end of the day (Project worker, area B)

### 3.3 Communication skills and interaction with project worker

Project workers' communication skills were valued by most participants. Many people felt they benefited from reflective listening and that this was an important part of the intervention:

It was very good and she … made me think of things that I never really thought about, and different things, which makes a difference if you've got an interviewer, doesn't it? … She was good, a good listener. (OP6, Female, 72 years)

I felt that I could talk to her, that I'd known her a long while. … and that she listened and suggested. (OP5, Female, 77 years)

Project workers thought that allowing time for a comprehensive first session was a key to building rapport with older people. They reported it was equally important when listening to people to both explore individual needs and act as a ‘change agent’ or a ‘life coach’.

The longitudinal nature of the intervention, delivered by the same project worker over a period of 6 months, facilitated a supportive and trusting relationship. The time when a participant felt comfortable in raising sensitive issues varied. A project worker gave an example:

Once we'd built up this trust, we then started talking about finances. (Project worker, area A)

### 3.4 Overall perceptions of goal setting and other BCTs

#### 3.4.1 Goal setting

Goal setting was talked about positively by most participants:

She used to say to me, ‘Could you go for a walk for half an hour?’ Then we increased it to three quarters of an hour and then we increased it to an hour. And I found it very therapeutic. (OP7, Female, 80 years)

However, project workers reported that it was not always easy for people to identify outcome goals. Some participants were able to generate goals in the first appointment, whereas others needed more time or guidance. Moreover, participants differed in how much they wanted the project worker to ‘push’ them to achieve their goals. Although most participants were happy with ‘gentle encouragement’, one said further pushing might have achieved a better outcome. Poor physical health was a barrier to progressing towards the main outcome goal in this example:

I suppose it could have been a bit more goal‐focused than it was. She never pushed me. She didn't put me under any pressure at all, which maybe she's not supposed to … and if I said I can't do something, she just sort of accepted it. (OP4, Male, 78 years)

Most participants felt a goal‐setting approach was relevant to their situation. However, two participants disliked the idea of working towards a goal and felt there was pressure to achieve them in the language used:

I was asked how would I like to be better? And, of course, there are ways I would like to be better than I am … But I felt then under pressure with letters coming, ‘You have undertaken to ...’ And I really objected to that. (OP11, Female, 82 years)

Project workers reported being careful to avoid using the language of behaviour change as they felt it might seem patronising. They found it helpful to reframe the idea of behaviour change to focus on maintenance of enjoyable behaviours:

I was quite clear at the start that I didn't want to ever say, it's a ‘change’ or ‘improve’. Because when I did, that was always a negative response. … [Instead I would say] ‘maintain,’ [and] always manage to get somebody engaged first. And then, once you set that maintain goal, then they're able to see, ‘Actually do I want to maintain the standard, do I want to improve it?’ And that's when you start to get goals for improvement for some people. (Project worker, area A)

#### 3.4.2 Monitoring and feedback on behaviour and outcomes

Most participants experienced monitoring progress towards goals and receiving feedback positively. As discussed above, this was a strong motivator but also triggered a proactive attitude towards different aspects of health and well‐being:

…when you haven't got that person, you just won't do it. You keep saying you're going to do it. But you don't, because you haven't got anyone to report back to, if you like, to say, ‘Well, yes I've done it’ … That was good in a way, that I had somebody to sort of report back to. (OP12, Female, 73 years)

Positive feedback from the project workers appeared to increase participants' motivation and facilitate progress towards their goals. Self‐monitoring, for example by keeping a food diary or by recording walking activity, was reported helpful by some:

…I felt that (project worker) was pleased that I was doing it. So it gave me more incentive to try and do a little bit more next time (OP6, Female, 72 years)

#### 3.4.3 Practical and emotional social support

Emotional support was important, especially to those living alone, those with mental health problems and those with complex needs (i.e. co‐existence of different needs, related to mental health, social circumstances, physical health), who found that having someone taking an interest in them contributed to better recovery from illness. The role of the project worker as a care coordinator was perceived as very helpful in addressing complicated problems including cognitive impairment, where practical social support was an essential part of the intervention:

…I was present for one of those appointments, and I know that the follow‐up from that was excellent. So, we were talking about possible help, getting a blue badge … And (project worker) got in touch with me about various things. (Family carer of OP14)

More examples of participants' experiences with BCTs and the context in which they were used are presented in Table 2.

**Table 2:** Examples of behaviour change techniques and context used

BCT	Context	Illustrative quotation
Goal setting (outcomes)	Mental well‐being	“…improve peace of mind – what is the priority for me – she asked me to improve peace of mind and reduce anxiety, decrease reliance on the medication, yes, yes.” (OP10, Female, 87 years)
	Physical activity	“It's enabled me to improve my energy wise, improve my walking, … the way my body was weak and all that, it's helped me with … the different exercises. It's helped me to move more, like I was not able to go up and down steps in the station there … she helped me to get more speedy.” (OP3, Female, 78 years)
Goal setting (behaviour)	Restructuring home environment, mental well‐being	“You're going to aim to sort out so many each time I come,” it made me get on and do it, gave me a push” (OP5, Female, 77 years)
Monitoring behaviour/outcomes	Physical activity	“She said, “What do you feel? Would this be better for you?” I said, “Yes, I want it stronger.” Then she brought me the things to, weight, well it's two, when I strap it on, I try to exercise with my feet, lift them up and down. And she constant sees me doing that. She asked to see me doing it, even though I marked it down that I did it. But she wants to see that. Yes.” (OP3, Female, 78 years)
Feedback on behaviour	Diet	“…she used to have a look at the book and see what's what and see how I was doing […] she would say, “You've done really well, you know, that's good,” and, you know, boosting me up saying, “You can do it.” (OP6, Female, 72 years)
Self‐monitoring of behaviour	Physical activity	“And she gave me the paper for me to record Monday I did this walk, Tuesday or Wednesday I did this walk.” (OP3, Female, 78 years)
	Diet	“Well we were talking about diet and things like that and (project worker) suggested doing a weekly planner of food for the week and see how I get on like that. So I did, I got myself a couple of books, one I put down the week before, the menu for the following week. And then I put down how I did it, did I stay to it, did I have a bad week? And that was quite good. That was something that I sort of still do now. I do think about, the week before, what we're going to have for next week and I'll sort it out.” (OP6, Female, 72 years)
Social support (practical)	Physical activity, memory	“(Project worker) looked into it. And that was, that was something she sent me through the post actually, was the different exercise classes that were available. And then we – we decided that that was the best one for my mum. So, my sister takes her there every Monday.” (Family carer of OP14)
Social support (emotional)	Mental well‐being	“She was encouraging, she was telling me that I was doing well, improving. It's very nice to be encouraged, but I was – actually when she was with me, I was well because she is so nice. And I was enjoying her visits.” (OP10, Female, 87 years)
Social support (practical and emotional)	Mental well‐being	“Well, first of all, she said about I shouldn't feel this guilt that I did, which, I mean when somebody tells you that, it does make you feel better. But she sort of encouraged me to, first of all, see a counsellor, which I did. Then she encouraged me to join things. And she gave me the information like the U3A, which I did join. So, that is how she helped me.” (OP12, Female, 73 years)
Habit formation	Diet	“You know, and I did it and once I started doing it, I got in the habit of doing it. But then I got better because I used to look back and then mark for like the week that had gone, what had I eaten on top of what I'd put down. You know, and things like that. So, as I say, I just got into the habit then of doing it, just took that little while to get really organised.” (OP6, Female, 72 years)
Social reward	Restructuring home environment, mental well‐being	“Well just, “Oh that's good, oh well done, you know, I'm really pleased and how do you feel about it?” you know. “Are you feeling that you're a bit more – less cluttered than you were?” Yes, encouragement. Praise for doing it.” (OP5, Female, 77 years)

## 4 DISCUSSION

A home‐based health promotion service for older people with mild frailty based on BCTs was overall well‐received by recipients. The majority was able to identify a range of goals to work on, related to mobility, physical activity and transport, socialising, mental well‐being, diet and finances. Most participants responded positively to goal setting, and only a few did not like the language or concept of setting goals. The process of goal setting could take time over several appointments. Most participants initially preferred the idea of setting goals to maintain their current independence/activities over the idea of improvement. Reflective listening and interaction with the same project worker over time enabled them to build a relationship of trust. Older people who had more complex needs reported benefiting from a combination of goal setting and elements of social, practical and emotional support (including helping co‐ordinate their care) in varying proportions, with involvement of a carer in some instances.

In our development work, qualitative interviews with older people, carers and health professionals revealed that a health promotion service should cover a broad range of domains, and use mechanisms such as providing information and signposting, emotional and practical support, and boosting motivation (Frost et al., 2017;2018 ). Although the COM‐B model (Michie et al., 2011), which underpinned the design of HomeHealth, has been increasingly used for behaviour change health promotion across different disciplines, few studies explore older people's experiences with this approach, and it is the first to focus on people who are mildly frail. In our study, capability clearly determined older people's choice of goals. Inherent capability acted as a substrate for external opportunity, both in the sense of maintaining physical function and promoting health, as well as dealing with problems. Although the conceptual framework of the development of the service was asset‐based (Morgan & Ziglio, 2007), the choice of goals was probably not completely free of a deficit‐oriented mentality, especially in older people whose physical health or cognitive functions had deteriorated recently. Opportunity was reported to increase as a result of receiving the service, through change of the physical environment, increasing social cues or combination of both physical and social triggers. Internal motivation, although hard for many to pinpoint, appeared to be strongly linked with the desire to live without the assistance of others. Interestingly, most participants were not sure if and how they would have experienced any change without the involvement of the project worker.

Our findings are comparable to those of a mixed‐methods study reporting on the use of BCTs to facilitate physical activity in older adults (Arnautovska, O'Callaghan, & Hamilton, 2017). The BCTs nominated as most useful by participants in that study were autonomy, information from a credible source, instruction to perform the behaviour, demonstration of the behaviour, self‐monitoring of behaviour, action planning, rewarding completion and information about health consequences. Monitoring of behaviour and giving feedback on behaviour and outcomes were felt to work well in our mildly frail population and helped maintain motivation. Our findings also demonstrate that other techniques such as practical and emotional social support are valuable for those with complicated health needs, including cognitive impairment. Both the positive receipt of feedback by most participants in our study and the emphasis on communication, leading to the establishment of a reciprocal, therapeutic relationship with the project worker, underline the importance of an intervention lasting some time. The importance of supportive relationships in engagement with behaviour change interventions has also been reported in younger populations (Sutcliffe et al., 2017).

The origin of motivation merits further exploration. Although the desire to remain independent was a commonly reported reason for entering the feasibility trial, most people emphasised the importance of the project worker acting as an external motivator, whose presence was key in introducing new ideas and creating a supportive framework to bring about change. Psychosocial support was also perceived as an important element of the intervention in a study exploring older people's experiences of a community matron primary care service (Williams, Smith, Chapman, & Oliver, 2011). Similarly, the importance of taking an interest in the older person was highlighted in a Swedish qualitative study about experiences of pre‐frail very old people who received preventive home visits to identify unmet needs and provide local service information (Dahlin‐Ivanoff et al., 2010). Interestingly, that study reported some people thought that such interventions were not for them because they were too ill or felt too old with nothing to anticipate (Behm, Ivanoff, & Zidén, 2013).

The level of need, defined by health and socioeconomic status, appears to be an important determinant of older people's engagement with health promotion. People who are ‘too fit’ may not engage with an intervention because they do not feel the need for doing so, whereas those with complex needs may struggle to engage because they face numerous barriers, leading to low levels of aspiration. In our study, older people's own subjective individual judgement about their needs was an important factor influencing their attitudes towards information provided by the project workers. Other research has shown that health maintenance‐related goals are the most common, whereas people with better health resources are more likely to report goals related to leisure‐time, social and physical activities, and those with poor social resources are at risk of having no personal goals (Saajanaho et al., 2016). These findings are in keeping with the theory of Ziegelmann and Knoll (2015) who distinguished health behaviours in two types: ‘proximal’, that is a core set of behaviours directly linked to physiological processes or producing straightforward health benefits, and ‘distal’, that is more complex activities indirectly linked to health‐related outcomes via different pathways. The hierarchy of goals is therefore inevitably shaped by the older person's perceptions of health and unmet needs. The reluctance of some participants in our study to identify themselves as being at risk of malnutrition can be explained by a different prioritisation of needs and desired outcomes from participation in a health promotion programme.

### 4.1 Strengths and limitations

The main strength of this qualitative study is the novelty of the findings. We found that older people with mild frailty can engage well with behaviour change interventions, providing they are tailored to their physical health needs, and delivered over time by a support worker with good communication skills. We interviewed both recipients and providers of the HomeHealth service, which gave complementary insights into factors affecting engagement. However, the data are drawn from a small sample participating in the feasibility trial, who as volunteers are likely to be more motivated. Additionally, not all agreed to be interviewed, so other views regarding behaviour change may be missing, for example half of the interviewees had a high level of education, which is associated with having more personal goals (Lawton, Moss, Winter, & Hoffman, 2002). Moreover, given the nature of the sampling, and having a small pool of participants in a feasibility study, it was not possible to use saturation as a method to ascertain completion of data collection. Only two participants were given a diagnosis of cognitive impairment during the delivery of the service, therefore findings for this group need to be interpreted with caution as the full range of views for this population is unlikely to have been explored.

### 4.2 Implications for practice and research

Results from this qualitative study are promising regarding the implementation of BCTs to promote health and well‐being in mildly frail older people. Capability to undertake change is a key aspect, and older people with mild frailty appear to benefit with tailored support from a service to address this. Further research is now needed to determine if this approach is both clinically and cost‐effective, before widespread implementation into routine care.

## 5 CONCLUSION

The majority of older people with mild frailty responded positively to a behaviour change approach delivered in the context of a home‐based health promotion service. Goal setting, monitoring of behaviour and feedback were perceived to increase motivation. Challenges of using goal setting in people with complex needs, including those with cognitive impairment, need to be accounted for when designing health promotion services. Practical and emotional social support to maximise capability should be included to promote health and well‐being in this group of older people who are becoming frailer.

## CONFLICT OF INTEREST

We have no conflict of interest to declare.
